# In memoriam—Professor Hugo Partsch (1938–2023)

**DOI:** 10.1111/iwj.14148

**Published:** 2023-03-21

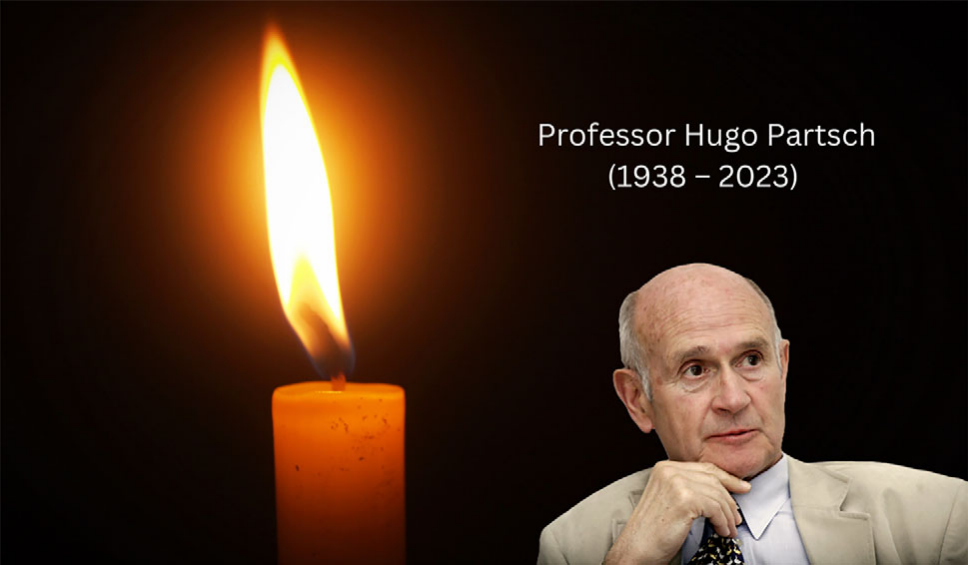

As long‐standing wound researchers, we were sad to hear of the passing of a wound‐care legend, Professor Hugo Partsch. Hugo for many is regarded as the grandfather of compression science. As an Austrian dermatologist, he contributed significantly to the research in phlebology and compression.

Here is his contribution to our journal in the 20 years of its existence and as a co‐author with our Editor‐in‐Chief:

- Harding, K. G., Vanscheidt, W., Partsch, H., Caprini, J. A., & Comerota, A. J. (2016). Adaptive compression therapy for venous leg ulcers: a clinically effective, patient‐centred approach. *International Wound Journal*, *13*(3), 317–325.
- Harding, K. G., Vanscheidt, W., Partsch, H., & Caprini, J. A. (2014). Actitouch System Improves Quality of Life; High Adherence to Therapy and High Satisfaction in Patients with Venous Leg Ulcers.
- Partsch, H. (2008). Intermittent pneumatic compression in immobile patients. *International Wound Journal*, *5*(3), 389–397.
- Flour, M., Clark, M., Partsch, H., Mosti, G., Uhl, J. F., Chauveau, M., … & Schingale, F. (2013). Dogmas and controversies in compression therapy: report of an International Compression Club (ICC) meeting, Brussels, May 2011. *International Wound Journal*, *10*(5), 516–526.
- Harding, K. G., Vanscheidt, W., Partsch, H., Caprini, J. A., & Comerota, A. J. (2016). Adaptive compression therapy for venous leg ulcers: a clinically effective, patient‐centred approach. *International Wound Journal*, *13*(3), 317–325.

However, his contribution was more significant that the short list above with his ResearchGate profile having more than 500 research items. In 2005, he founded the International Compression Club (ICC), a forum that links medical experts with industry and aims to promote the science behind compression therapy.

Personal reflection (DQ): In 2008, I had the honour of meeting Hugo for the first and only time in my long wound‐care career. He was presented with a Lifetime Achievement Award for his contribution to wound care. This was the citation of his award—“Professor Partsch is the Co‐Editor of *VASA, Journal for Vascular Diseases*. A former Professor of Dermatology at the University of Vienna and the former Head of the Dermatological Department at the Wilhelminen‐Hospital (Vienna), he has published more than 360 papers in scientific medical journals and contributed to books.” Witnessing him receiving this award among the “best of the best” in wound care cemented his “rock star” status in my mind.

Personal reflection (KGH)—Hugo was an excellent clinician, a global leader in research into leg ulcers, an excellent and inspiring educator but also was a true gentleman. He always had time to talk and put you in contact with people. He was admired and respected by all he met. Even in his latter years, when we met up at conferences he would be the first to appear for breakfast—usually after an early morning run—and would be a fantastic source of ideas, contacts, and data. I was fortunate to have the honour to publish with Hugo as a co‐author and without doubt, it was one of the privileges and highlights of my academic career.

His passing while sad reminds us all of the extensive influence of Hugo Partsch in the wound‐care area and he will leave a legacy in this area for other clinicians and scientists to benefit from. His legacy will live on through the continued work of many others.

Keith Harding, Editor‐in‐Chief, IWJ

Douglas Queen, Editor, IWJ

